# Switchable Photonic Nanojet by Electro-Switching Nematic Liquid Crystals

**DOI:** 10.3390/nano9010072

**Published:** 2019-01-06

**Authors:** Bintao Du, Jun Xia, Jun Wu, Jian Zhao, Hao Zhang

**Affiliations:** Joint International Research Laboratory of Information Display and Visualization, School of Electronic Science and Engineering, Southeast University, Nanjing 210096, China; 230179712@seu.edu.cn (B.D.); zj616@hotmail.com (J.Z.); haozhang_seu@hotmail.com (H.Z.)

**Keywords:** micro-optics, photonic nanojet, nanomaterials

## Abstract

This paper first presents a switchable photonic nanojet (PNJ) generated by a polystyrene (PS) microsphere immersed in nematic liquid crystals (NLCs). The PNJ is switched by applying external voltage, which originates from the refractive index change in the surrounding medium caused by the field-induced realignment of liquid crystal molecules. By tuning the refractive index of NLCs larger or smaller than that of the PS microsphere, the PNJ can be switched off or on. Moreover, we present an optimization study to seek a better electric energy focusing property of the PNJ. Our results reveal that the switchability of PNJ can be optimized by applying a shorter incident wavelength, a double-layer microsphere, and a PS ellipsoid. The full width at half-maximum (FWHM) generated by the PS ellipsoid is narrower than that generated by the microsphere with a shorter incident wavelength. The intensity contrast of the PS ellipsoid is higher than that of the double-layer microsphere. As a whole, the switchability of PNJ can be best optimized by a PS ellipsoid. This should open the way for the development of integrated photonic devices.

## 1. Introduction

Sub-wavelength optical resolution has become essential for various applications, from optical microscopy [1], lithography [2], and spectroscopy [3], to data storage [4]. Obtaining the finest resolution [5,6] has been a hot topic for a long time. However, the conventional objective lens has diffraction-limited optical spots [7]. Much effort has been devoted to finding a new way to focus the light to a small spot beyond the diffraction limit [7,8]. The super-focusing effect of “photonic nanojets (PNJs)” [9,10,11,12,13,14,15,16,17,18,19,20,21] produced by a dielectric microsphere or microcylinder emerged as a simple and effective way to break the diffraction limit. The PNJ is a narrow high-intensity light flow generated from the shadow surface of transparent nanoparticles. When a transparent nanoparticle with a dimension larger than the wavelength is illuminated by light, PNJ is created due to interferences between the illuminating field and scattering field. The most striking and distinctive feature of PNJ is the high spatial localization of light field in the transverse direction (relative to the direction of incidence), which, in contrast to the conventional high-NA (numerical aperture) focusing optics, can lead to super-resolution dimensions. Moreover, the maximum intensity of the PNJ can reach several hundred times compared to that of the incident light. These features bring about the potential applications of PNJ for nanoscale processing of materials [22], high-resolution microscopy [23], localized sensing techniques [24], optical data storage [25], optical antennas [26], and so on.

It has been suggested that the refractive index contrast between the nanoparticle and its background medium plays an important role in the characters of PNJ [27]. Thus, tuning the refractive index of the background medium by external stimuli becomes a key to manipulating PNJ dynamically. Recently, various kinds of micron-sized optical functional materials and devices using liquid crystal (LC) microdroplets have been proposed [28,29,30,31,32]. Primarily, the generation of a PNJ from an LC microcylinder with tangential molecular alignment was theoretically analyzed by finite-difference time-domain (FDTD) [28]. Later on, direct experimental observation of electrically tunable PNJ generated from self-assembled LC microdroplets was reported [29]. Such tunable PNJ was also achieved by using a shell and LC core architecture [30]. Similar tunable PNJs created by a core-shell microcylinder with nematic LCs infiltrated were presented in References [31,32]. These results indicate that the LC is a good choice for PNJ manipulation due to its rich variety of molecular alignments.

Unlike the previous studies above, in this paper, we present a study on a switchable PNJ generated by a polystyrene (PS) microsphere surrounded by nematic liquid crystals (NLCs). Applying an electric field to the device alters the directors of LC molecules, which changes the effective refractive index, and electro-switching of PNJ is realized. FDTD simulation and analysis results show that the PNJ can be switched on and off by modulating the refractive index of NLCs smaller and larger than that of the PS microsphere. This is the first time, to the best of our knowledge, that such a case is reported in the literature. Furthermore, optimization PNJ switching by different methods are studied in an ideal model. The results reveal the potential of micro-sized particles immersed in the LCs to control light beyond the diffraction limit. This can be of interest for several applications, especially in optoelectronic devices. 

## 2. Influence of Surrounding Medium on PNJ

Several calculation methods have theoretically and numerically analyzed electromagnetic field distribution in the vicinity of a transparent nanoparticle illuminated by a plane wave [9,27]. These calculations show that the background medium surrounding nanoparticles plays a significant role in the formation of a PNJ. To verify the effect of the surrounding medium, we performed FDTD simulations using commercial Lumerical Solutions software.

**Figure 1:**
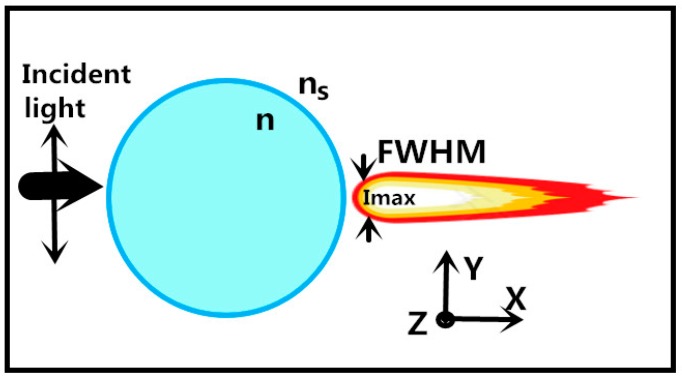
Schematic diagram of a photonic nanojet (PNJ) produced by a dielectric microsphere.

The schematic diagram of a PNJ produced by a dielectric microsphere is shown in Figure 1. The narrow, high-intensity PNJ that emerges from a dielectric microsphere has been found to present several key properties. First, the maximum intensity of PNJ is several hundred times higher compared to that of the incident light. Second, the full width at half-maximum (FWHM) of PNJ can be less than the classical diffraction limit. In our simulation, incident beams are linearly polarized with a wavelength of 540 nm, and a 5 μm-diameter barium titanate glass (BTG) microsphere with a refractive index of 1.9 is placed in a medium with refractive index of *n*_s_.

**Figure 2:**
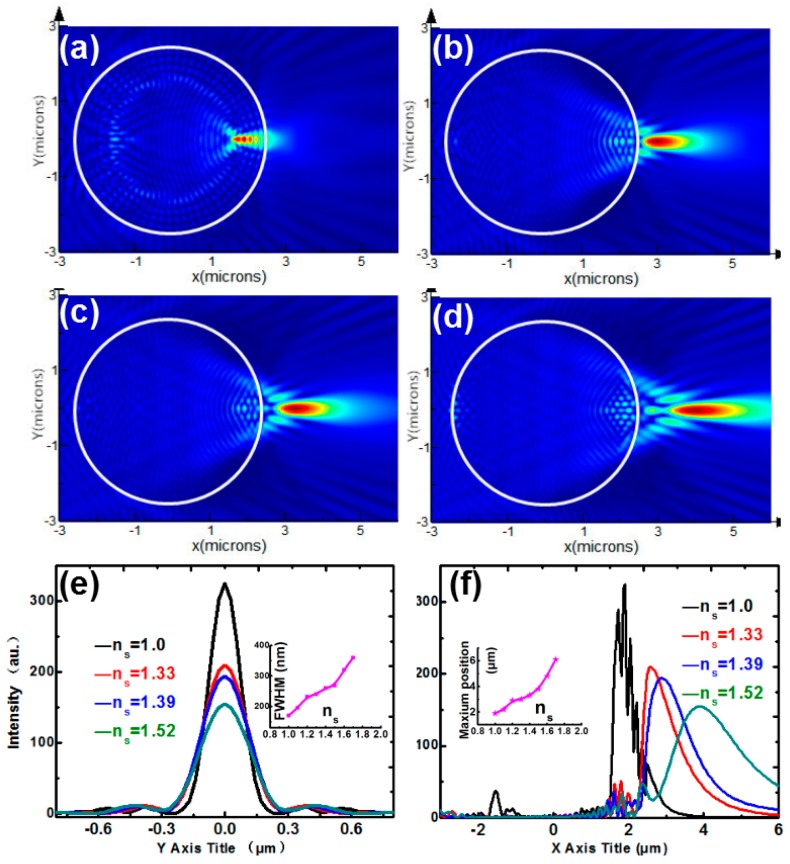
Intensity distributions of barium titanate glass (BTG) microspheres immersed in different surrounding medium for (**a**) *n*_s_ = 1.0, (**b**) *n*_s_ = 1.33, (**c**) *n*_s_ = 1.39, (**d**) *n*_s_ = 1.52. (**e**) Transverse intensity profiles for *n*_s_ = 1.0, 1.33, 1.39, and 1.52 at maximum intensity spots. (**f**) Longitudinal intensity profiles for *n*_s_ = 1.0, 1.33, 1.39, and 1.52 sliced along *Y* = 0. The insets of (**e**,**f**) correspond to the FWHM and peak intensity position as a function of n_s_.

Figure 2a–d show the intensity distributions of BTG microspheres immersed by different surrounding medium for *n*_s_ = 1.0, 1.33, 1.39, 1.52, respectively. We could see that the PNJ will gradually shift from the inside to the outside of microspheres when the surrounding medium refractive index n_s_ increases from 1.0 to 1.52. According to Figure 2f, the maximum intensity position of the PNJ increases from 1.9 μm to 3.8 μm as the surrounding refractive index increases. In Figure 2e, the FWHMs are 170, 246, 260, and 272 nm corresponding to *n*_s_ = 1.0, 1.33, 1.39, and 1.52, respectively. It indicates that the FWHM of the PNJ monotonically increases with the growth of the refractive index of the surrounding medium as well. The insets in Figure 2e,f show the evolution of the FWHM with the maximum intensity position with n_s_ increasing from 1.0 to 1.52. Evidently, increasing *n*_s_ results in elongated PNJ, accompanied by decreased peak intensity and broadens the FWHM. Figure 2 is in agreement with the tendency studied in previous works, which indicates that the key parameter of the PNJ depends significantly on the refractive index of the surrounding medium. Therefore, the key to manipulate PNJ is to find a material with a variable refractive index. 

## 3. Birefringence of Nematic Liquid Crystals (LNCs)

Nematic liquid crystals (NLCs) generally have positive optical anisotropy, and the major axis of NLC molecules can be orientationally aligned into the direction decided by the external voltage and confining surfaces [33]. If an external electric voltage is applied to the NLCs confined by two surfaces, the molecules of NLCs tend to rotate to the direction parallel to the external electric field. The molecules of NLCs also return to the original configuration once the electric field is switched off, originating from the restoring torques provided by the strong anchoring on the confining surface of the device. Due to the high birefringence and sensitive response to the external stress, NLCs become a good candidate for PNJ manipulation. 

The NLC studied in this paper is positive uniaxial. If we denote the crystalline axes by *rx*, *ry*, and *rz*, the refractive indexes are *n_rx_*, *n_ry_*, and *n_rz_*, for light polarized along *rx*, *ry*, and *rz*, respectively. As they are positive uniaxial, it can be assumed that *n_rx_* = *n_ry_* = *n*_o_, *n_rz_* = *n*_e_, where *n*_o_ is the refractive index along minor axis and *n*_e_ is that along the major axis. The refractive indexes along the three axles of the NLC molecule can be characterized by [33]:

(1) $$n_{rx}=\sqrt{\varepsilon_{rx}},n_{ry}=\sqrt{\varepsilon_{ry}},n_{rz}=\sqrt{\varepsilon_{rz}}.$$

where *ɛ_rx_* and *ɛ_ry_* are the ordinary dielectric constants and *ɛ_rz_* is the extraordinary dielectric constant. According to Maxwell’s equations, Fresnel Equation is given by the next equation.

(2) $$\frac{k_{rx}^{2}}{\frac{1}{n^{2}}-\frac{1}{\varepsilon_{rx}}}+\frac{k_{ry}^{2}}{\frac{1}{n^{2}}-\frac{1}{\varepsilon_{ry}}}+\frac{k_{rz}^{2}}{\frac{1}{n^{2}}-\frac{1}{\varepsilon_{rz}}}=0.$$

**Figure 3:**
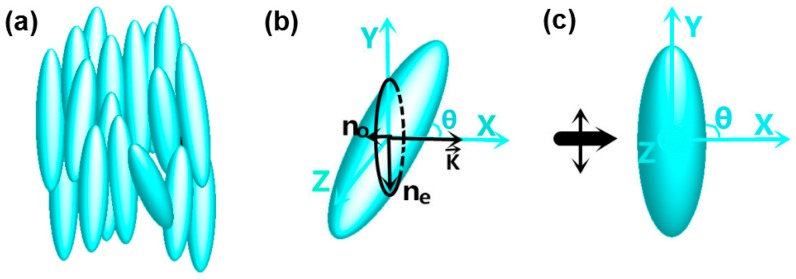
(**a**) Schematic view of the arrangement of nematic liquid crystals (NLC) molecules. (**b**) Index ellipsoid method for a polarized incident light passing through a uniaxial crystal. *n*_o_ and *n*_e_ correspond to the refractive indexes for the ordinary and extraordinary components, respectively. (**c**) Nematic liquid crystal molecule rotating in *X*–*Y* plane with incident light polarized along Y axis.

The solution of the Equation (2) is described as:

(3) $$\left\{ \begin{array}{l} n_{1}=n_{o}\\ n_{2}=n_{e}(\theta )=\frac{n_{o}n_{e}}{\sqrt{n_{o}^{2}\sin^{2}\theta +n_{e}^{2}\cos^{2}\theta }} \end{array}\right.$$

where *θ* is the rotation angle between light propagating direction and the NLC molecule. This means that the lightwave traversing through NLC generally possess two refractive indexes. One is *n*_o_, the other is *n*_e_. For light of arbitrary polarizations, or equivalently, an electro-optics crystal of an arbitrary orientation relative to the polarized light propagation, the resulting propagation of polarized light in the crystal is rather complex [34]. We perform the theoretical analysis of light propagation in NLCs by index ellipsoid method for simplicity [34]. For a linearly polarized light, if the angle between the propagating direction *k* and the NLC molecule is *θ*, the cross-section of the NLC molecule and the plane perpendicular to *k* is an ellipse, as shown in Figure 3b. According to the index ellipsoid method, the major axis of ellipse equals to the value *n*_e_, and the minor axis of ellipse equals to the value *n*_o_. The lightwave, whose electric field oscillates along *n*_e_ vector direction, senses a larger refractive index, corresponding to the extraordinary index *n*_e_(*θ*). The lightwave, whose electric field oscillates along *n*_o_ vector direction, senses the ordinary index, which is a constant *n*_o_. The intensities of the ordinary and extraordinary light are determined by the components of the initial intensity along *n*_o_ vector and *n*_e_ vector, respectively.

If the NLC molecules rotate in *X*–*Y* plane (as shown in Figure 3b), the incident light, which is arbitrary linearly polarized, can be decomposed into two components along *n*_e_ and *n*_o_ vector respectively. This means that only the light decomposed along *n*_e_ vector can sense the refractive index change from *n*_e_(*θ*) to *n*_o_, leading to a low contrast during a tunable process. The light decomposed along *n*_o_ vector senses unchanged refractive index *n*_o_ before and after tuning, which looks like background noise. If the incident light is polarized along *n*_e_ vector as shown in Figure 3c, the extraordinary light component is maximum, while the ordinary light component is minimum. It is the best result we want. So, an incident light polarized along *Y*-axis is chosen in this paper.

## 4. Materials and Methods

**Figure 4:**
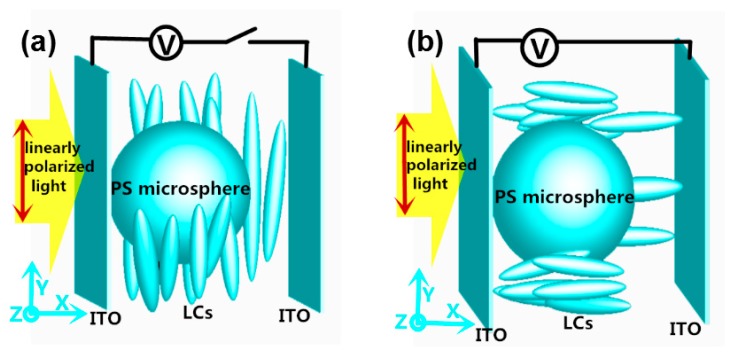
The arrangements of LCs (**a**) with an external voltage switched off; (**b**) with an external voltage switched on.

To study the switchability of the microsphere though its surrounding medium, we consider a 5-μm-diameter polystyrene (PS) microsphere with a constant refractive index *n* = 1.62 immersed in the NLCs with a tunable refractive index *n*_s_. The NLC is sandwiched between indium tin (ITO) layers. The inner surface of ITO is coated with polyimide and unidirectionally rubbed along *Y*-axis. The incident plane light is *Y*-axis polarized, and the wavelength is 540 nm. To study the evolution of PNJ within a sufficient perspective, the whole size of NLCs in this paper is several micrometers away from the PS microsphere, as shown in Figure 4.

When a microsphere is immersed in the NLCs, the director field is always distorted even if the surface anchoring of nematic molecules on the particle is weak. Here, unidirectional planar anchoring is assumed for both planar surfaces and PS microsphere. The boundary conditions are met by the creation of two surface defects, called boojums, located at the poles of the microsphere [35,36]. Actually, by contrast to the microsphere body, the light traveling through the poles of the microsphere plays a weak role in PNJ formation. Surface defect is an impact factor on PNJ control, but not a decisive factor any more. It is difficult to simulate a boojums configuration around the PS microsphere, and an ideal model is used in the following simulation. In the ideal model, the boojums configuration and the ITO layer are negligible.

As shown in Figure 4a, in the absence of an external voltage, the major molecular axis of the LC is aligned parallel to the substrates, corresponding to an effective index of 1.75 for extraordinary light. In contrast, when voltage is introduced, the LC molecules start to change their direction towards the *X*-axis, resulting in a refractive index of 1.52 for extraordinary light. By tuning the effective refractive index larger or smaller than that of the PS microsphere, the PNJ can be switched off and on.

## 5. Results and Discussion

**Figure 5:**
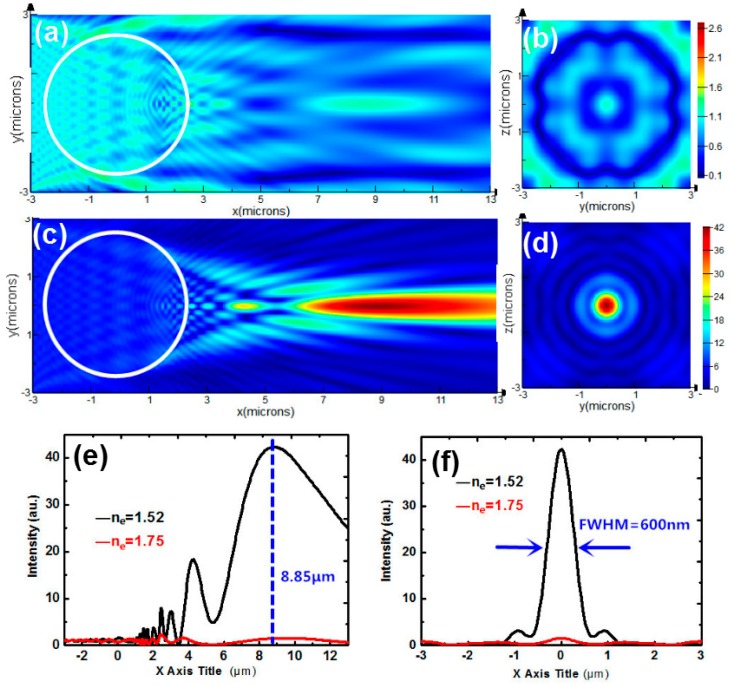
Intensity distribution of a polystyrene (PS) microsphere immersed by NLCs for (**a**) *n*_e_ = 1.75 (**c**) *n*_e_ = 1.52. (**b**,**d**) Transverse intensity profiles sliced along *X* = 8.85 μm corresponding to (**a**,**c**). (**e**) Longitudinal intensity graphs for *n*_e_ = 1.52 (black curve) and 1.75 (red curve) sliced along *Y* = 0. (**f**) Transverse intensity graphs for *n*_e_ = 1.52 (black curve) and 1.75 (red curve) sliced along *X* = 8.85 μm.

When *n*_e_ changes from 1.75 to 1.52, the spatial distribution of PNJ also changes, as shown in Figure 5a,c. Figure 5b,d correspond to the cross-sections of Figure 5a,c at the peak intensity, which are sliced along *X* = 8.85 μm. The longitudinal intensity graphs shown in Figure 5e is sliced along *Y* = 0. In Figure 5f, the transverse FWHM of nanojet is measured to be 600 nm. By switching the refractive index of extraordinary light from 1.75 to 1.52, the intensity at *X* = 8.85 μm changes from 1.5 to 42. To study the switchability of photonic nanojet, it is necessary to define an intensity contrast. The intensity contrast is given by the ratio between the peak intensity for *n*_e_ = 1.52 and the intensity at the same position for *n*_e_ = 1.75. And the intensity contrast here is 28. Applying an electric field to the device adjusts the director of the LCs, which changes the effective refractive index, and the electro-switching of PNJ is realized. However, the cost for this switching of photonic nanojet is widening the FWHM and reducing the peak intensity. Based on the application of active tuning in nanophotonics, there is a need to optimize the PNJ switchability of this technique. We, therefore, present an optimization study which seeks better electric energy focusing properties of PNJ.

**Figure 6:**
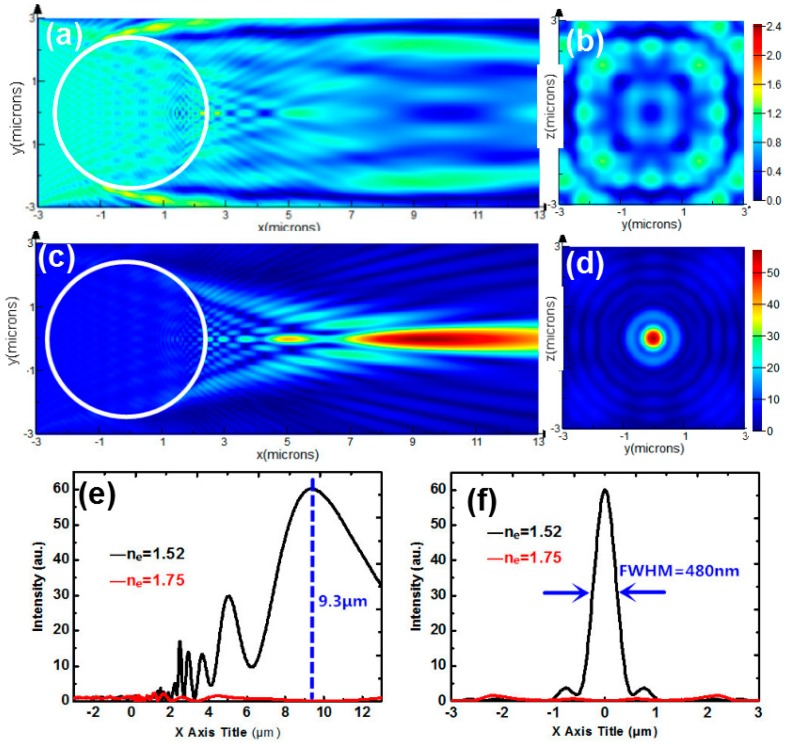
(**a**) Intensity distribution of a PS microsphere with a shorter incident wavelength of 400 nm. for (**a**) *n*_e_ = 1.75 (**c**) *n*_e_ = 1.52. (**b**,**d**) Transverse intensity profiles sliced along *X* = 9.3 μm corresponding to (**a**,**c**). (**e**) Longitudinal intensity graphs for *n_e_* = 1.52 (black curve) and 1.75 (red curve) sliced along *Y* = 0. (**f**) Transverse intensity graphs for *n*_e_ = 1.52 (black curve) and 1.75 (red curve) sliced along *X* = 9.3 μm.

The same structure but with a shorter incident wavelength of 400 nm is analyzed and discussed first. In fact, NLCs with different incident wavelengths have different birefringence due to dispersion. However, the difference of ∆*n* (*n*_e_(*θ*) − *n*_o_) is only about 0.05 here, which plays a weak role in the switching of PNJ. So, the dispersion in our study is negligible. Figure 6a,c show the spatial distributions of PNJs for *n*_e_ = 1.75 and 1.52, respectively. Figure 6b,d are the corresponding cross sections sliced along *X* = 9.3 μm. It can be seen that the PS microsphere with 400 nm incident wavelength provides an FWHM of 480 nm, which is tighter compared to that with 540 nm incident wavelength, as shown in Figure 6f. However, the peak position shifts from 8.85 μm to 9.3 μm when the incident wavelength decreases from 540 nm to 400 nm. By switching the refractive index of extraordinary light from 1.75 to 1.52, the intensity at *X* = 8.85 μm changes from 0.3 to 60. The intensity contrast in Figure 6 is 200, obviously stronger than that shown in Figure 5. The results showed that the switching properties of PNJ can be optimized by tuning the wavelength of the incident light to a shorter one.

**Figure 7:**
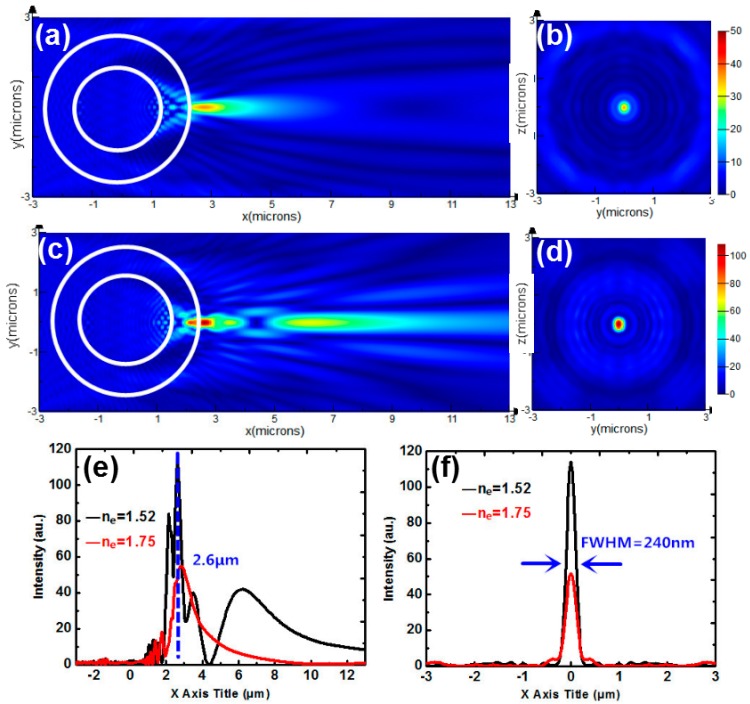
(**a**) Intensity distribution of a coupled core-shell microsphere immersed by NLCs for (**a**) *n*_e_ = 1.75 (**c**) *n*_e_ = 1.52. (**b**,**d**) Transverse intensity profiles sliced along *X* = 2.6 μm corresponding to (**a**,**c**). (**e**) Longitudinal intensity graphs for *n*_e_ = 1.52 (black curve) and 1.75 (red curve) sliced along *Y* = 0. (**f**) Transverse intensity graphs for *n*_e_ = 1.52 (black curve) and 1.75 (red curve) sliced along *X* = 2.6 μm.

Next, we study a microsphere consisting of a BTG spherical core with a refractive index of 1.9 coated with a PS concentric shell. The diameter of the core is 3 μm, and the thickness of the shell is 1 μm, as shown in Figure 7. Figure 7a,c show the intensity distributions of the coupled core-shell microsphere for *n*_e_ = 1.75 and 1.52, respectively. Figure 7b,d correspond to the transverse intensity profiles of Figure 7a,c sliced along *X* = 2.6 μm. Longitudinal intensity graph sliced along *Y* = 0 is shown in Figure 7e. The FWHM for *n*_e_ = 1.52 in Figure 7f is 240 nm. The focusing of the optical field by such a coupled core-shell microsphere becomes narrower compared to that generated by a one-layered homogeneous sphere in Figure 5 or Figure 6. However, the intensities for *n*_e_ = 1.52 and 1.75 at *X* = 2.6 μm are 114 and 52, respectively, resulting in a low-intensity contrast of 2.2. So, the coupled core-shell microsphere leads to a narrow FWHM but accompanied with a decreased intensity contrast.

**Figure 8:**
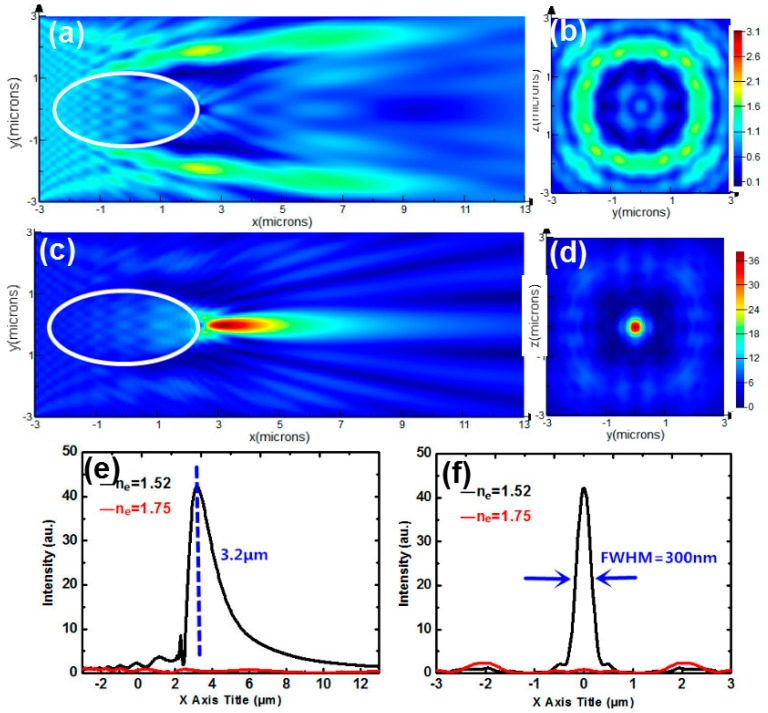
(**a**) Intensity distribution of micron-scale spheroid immersed by NLCs for (**a**) *n*_e_ = 1.75 (**c**) *n*_e_ = 1.52. (**b**,**d**) Transverse intensity profiles sliced along *X* = 3.2 μm corresponding to (**a**,**c**). (**e**) Longitudinal intensity graphs for *n*_e_ = 1.52 (black curve) and 1.75 (red curve) sliced along *Y* = 0. (**f**) Transverse intensity graphs for *n*_e_ = 1.52 (black curve) and 1.75 (red curve) sliced along *X* = 3.2 μm.

We further study the spatial distribution of the intensity produced by a spheroid. The corresponding major and minor diameters are 5 μm and 2.5 μm, respectively. Figure 8a,c show the intensity distributions of a spheroid immersed by NLCs for *n*_e_ = 1.75 and *n*_e_ = 1.52. Figure 8b,d show the transverse intensity profiles sliced along *X* = 3.2 μm. The intensity for *n_e_* = 1.52 reaches the maximum 42 at *X* = 3.2 μm, while intensity for *n*_e_ = 1.75 is only 0.8. The intensity contrast is about 52 and the FWHM shown in Figure 8f is 300 nm. It turns out that the FWHM with the oblate spheroid is narrower than that with the spherical one in Figure 5 or Figure 6. Meanwhile, the intensity contrast here is higher than that in Figure 5 and Figure 7. Therefore, the switchable photonic nanojet can be approximated with high efficiency by such a spheroid. 

These studies reveal that the switchability of the PNJ can be optimized by applying a shorter incident wavelength, a double-layer microsphere or a PS ellipsoid, respectively. The FWHM generated by the PS ellipsoid is narrower than that generated by the microsphere with shorter incident wavelength. The intensity contrast of such PS ellipsoid is also higher than that of the double-layer microsphere. We conclude that the switchability of PNJ can be best optimized by an ellipsoid in an ideal model. Although there had been many studies for tuning the PNJ properties using different size, material, geometry of spheres or core-shell spheres [37,38,39], they are an almost passive tunable process. Once the structures are designed and fabricated, their PNJ properties cannot be changed. The main difference here is that our method allows for dynamic tunability using electrical control in real time. We pay more attention to the PNJ change for the NLC effective refractive index larger and smaller than that of the PS microsphere. In practice, natural materials with such relationship are infrequent and finding other possible materials is a deserved research for further study.

## 6. Conclusions

In this paper, a switchable photonic nanojet achieved by a PS microsphere with NLCs immersed is reported. The effective refractive index of NLCs can be changed by tuning the alignment of the liquid crystal molecules. Therefore, we are able to switch the photonic nanojet by tuning the surrounding refractive index. Using high-resolution FDTD simulation, we indicate that the photonic nanojet is dynamically switched by a PS microsphere immersed in the NLCs. Moreover, we present an optimization study which seeks better electric energy focusing properties of the PNJ. As a whole, the switchability of the photonic nanojet can be best optimized by an ellipsoid. Such a mechanism for nanojet manipulation may open up new areas for a photonic device with subwavelength spatial resolution.
